# Supplementary material for: Robust trigger wave speed in Xenopus cytoplasmic extracts
Source: Nat Commun. 2024 Jul 10;15:5782. doi: 10.1038/s41467-024-50119-0 (PMC11237086; doi:10.1038/s41467-024-50119-0)
Supplement: Supplementary file 1 — Supplementary Information [file 41467_2024_50119_MOESM1_ESM.pdf]

Supplementary Materials for

Robust trigger wave speed in *Xenopus* cytoplasmic extracts

Jo-Hsi Huang, Yuping Chen, William Y. C. Huang, Saman Tabatabaee, and James E. Ferrell Jr.

Correspondence to: johsi@stanford.edu and james.ferrell@stanford.edu

This PDF file includes:

Supplementary Figures

Supplementary Table

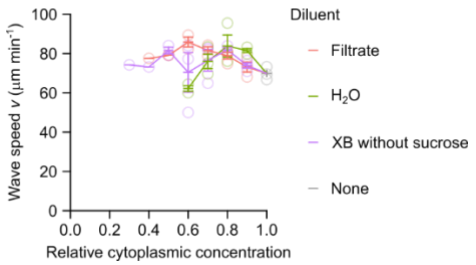

Supplementary Fig. 1. Mitotic trigger wave speed is robust to dilution using either filtrate or buffer.

Speeds of mitotic trigger waves at different cytoplasmic concentrations. The cycling extracts were diluted using either filtrate, XB buffer without sucrose, or water. Note that dilution with filtrate and buffer produced comparable wave speeds, whereas dilution with water resulted in a drop in speed. For extracts diluted with water below 0.6x, cycle progression was not observed. Means ± S.E.M. compiled from n = 3 independent samples are shown.

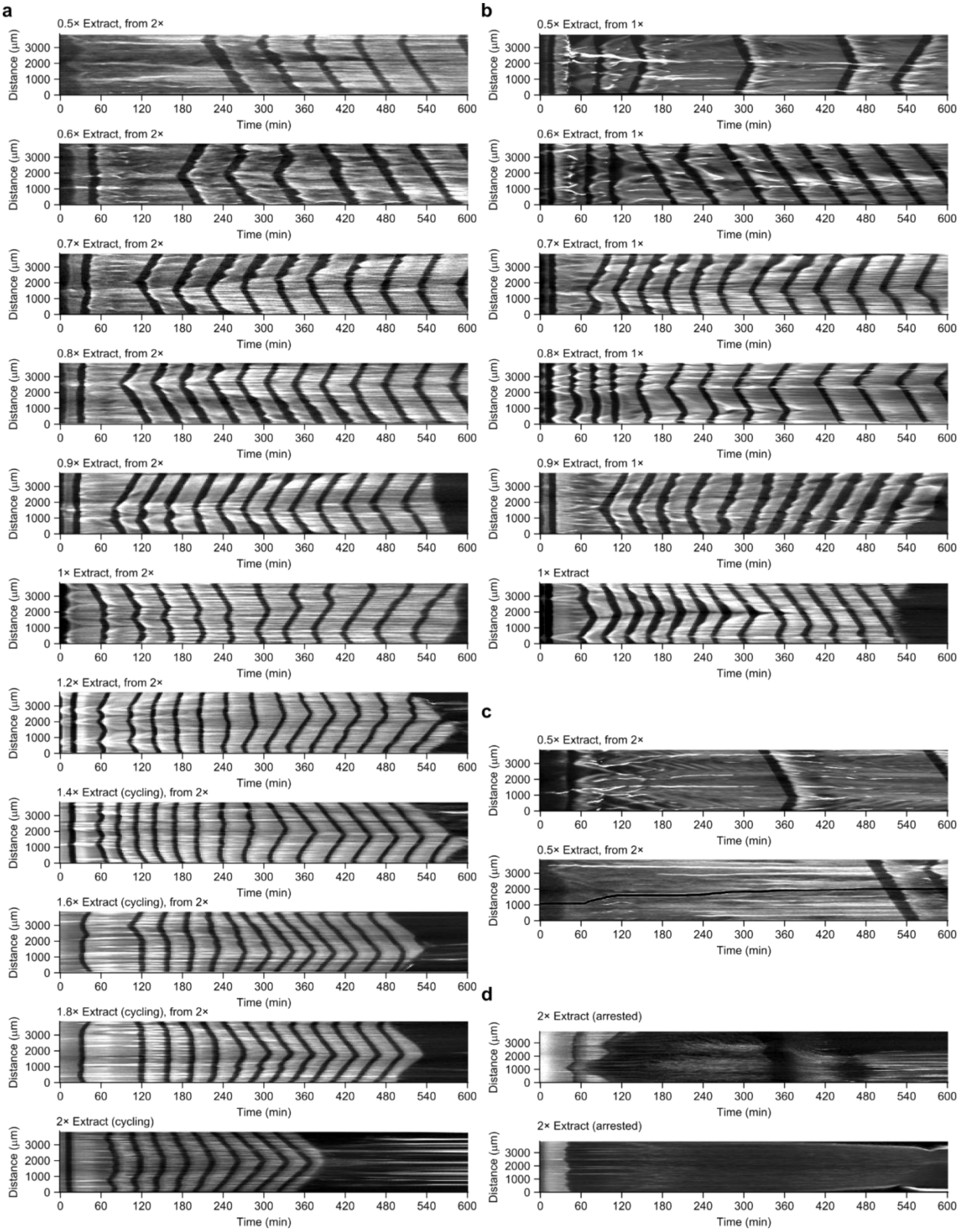

a
0.5× Extract, from 2×
0.6× Extract, from 2×
0.7× Extract, from 2×
0.8× Extract, from 2×
0.9× Extract, from 2×
1× Extract, from 2×
1.2× Extract, from 2×
1.4× Extract (cycling), from 2×
1.6× Extract (cycling), from 2×
1.8× Extract (cycling), from 2×
2× Extract (cycling)
b
0.5× Extract, from 1×
0.6× Extract, from 1×
0.7× Extract, from 1×
0.8× Extract, from 1×
0.9× Extract, from 1×
1× Extract
c
0.5× Extract, from 2×
0.5× Extract, from 2×
d
2× Extract (arrested)
2× Extract (arrested)
Distance (μm)
0
1000
2000
3000
Time (min)
0
60
120
180
240
300
360
420
480
540
600

Supplementary Fig. 2. Cell cycle and mitotic trigger waves at different cytoplasmic concentrations.

a, **b** Representative kymographs depict cell cycle and mitotic trigger waves at various cytoplasmic concentrations, prepared from 2x retentate (**a**) and 1x extract (**b**). **c** Two additional instances showcase 0.5x extracts prepared from 2x retentate, highlighting the variability in the first complete cell cycle across different extracts. **d** In contrast to the continuous cycling observed in Fig. 1e, these two examples of 2x retentate underwent mitotic arrest, occurring either in the first mitosis or the second.

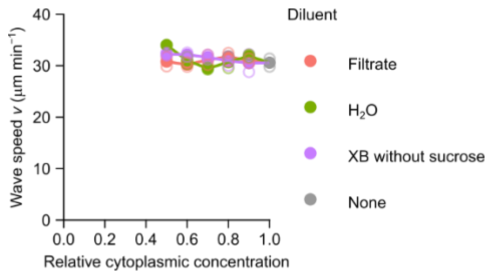

Supplementary Fig. 3. Apoptotic trigger wave speed is robust to dilution using filtrate, buffer, and water.

Speeds of apoptotic trigger wave at different cytoplasmic concentrations. The interphase extracts were diluted using either filtrate, XB buffer without sucrose, or water. Means from n = 2 independent experiments are shown.

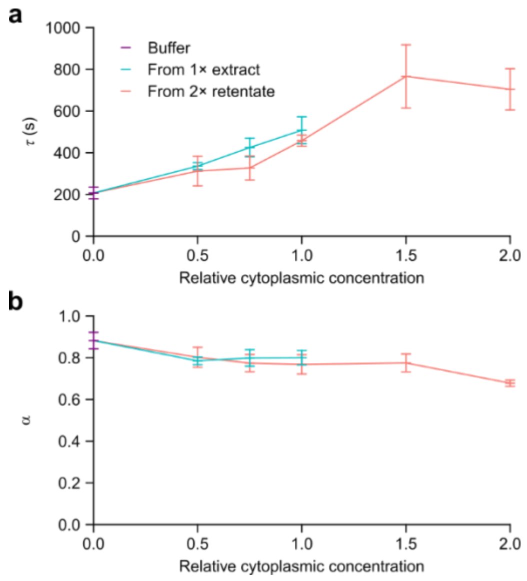

Supplementary Fig. 4. Anomalous diffusion fit to AF488-BSA diffusion in extracts.

a, **b** FCS autocorrelation functions of AF488-BSA in extracts analyzed using the anomalous diffusion framework. Original data is the same as in Figure 4. Mean ± 90 CI from 3 measurements are shown (n = 1). Diffusion time τ_D (**a**) and anomalous diffusion exponent α (**b**) at different cytoplasmic concentrations.

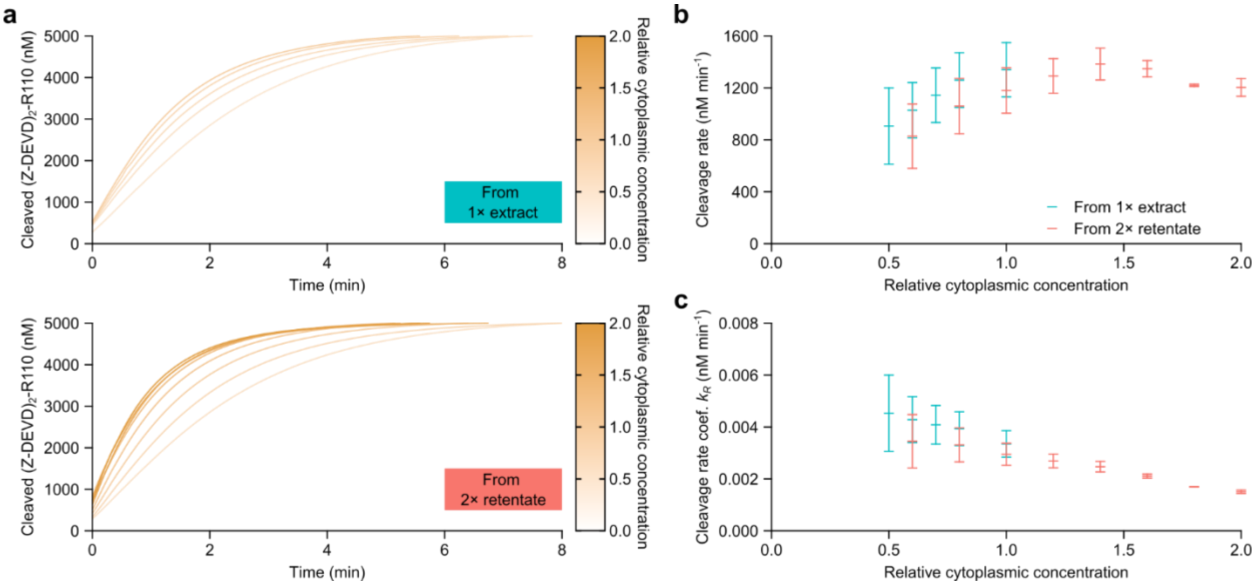

Supplementary Fig. 5. Measuring (Z-DEVD)2-R110 cleavage rate coefficient k_R.

a Kinetics of (Z-DEVD)2-R110 cleavage in freshly prepared apoptotic extracts at various cytoplasmic concentration. These extracts were prepared from 1x extract (top) or 2x retentate (bottom). Data from 1 experiment are shown. **b** Cleavage rates of (Z-DEVD)2-R110 at different cytoplasmic concentrations were estimated based on the initial timepoints. **c** Second-order rate coefficients at different cytoplasmic concentrations were computed by accounting for (Z-DEVD)2-R110 concentration and nominal active caspase 3/7 concentrations. Data are presented as means ± S.E.M. compiled from n = 3 experiments.

Supplementary Table 1. List of parameters used in caspase 3/7 activation kinetics inference.

Name	Meaning	Value	Unit	Source
R_{total}	Total concentration of $(Z\text{-}DEVD)_2$-R110 and R110	2000	nM	Nominal
R_0	Initial concentration of Rhodamine 110		nM	Measured
C_{total}	Total concentration of inactive and active caspase 3/7	100 – 400	nM	Nominal
C_0	Initial concentration of active caspase 3/7		nM	Fitted
k_R	Rate constant for $(Z\text{-}DEVD)_2$-R110 cleavage by caspase 3/7		nM^{-1} min^{-1}	Measured
k	Rate constant for caspase 3/7 activation		nM^{-1} min^{-1}	Fitted
k_{BG}	Rate constant for nonspecific cleavage of $(Z\text{-}DEVD)_2$-R110		min^{-1}	Fitted
